# Highly Reliable Method to Obtain a Correlation Coefficient Unaffected by the SEM Noise Component for Examining the Degree of Specimen Damage due to Electron Beam Irradiation

**DOI:** 10.1155/2021/2226577

**Published:** 2021-11-15

**Authors:** Eisaku Oho, Kazuhiko Suzuki, Sadao Yamazaki

**Affiliations:** ^1^Department of Electrical and Electronic Engineering, Faculty of Engineering, Kogakuin University, 2665-1 Nakano-Machi, Hachioji, Tokyo 192-0015, Japan; ^2^Research & Development Center, Nohmi Bosai Ltd., 1-18-13, Chuo, Misato, Saitama 341-0038, Japan

## Abstract

A correlation coefficient is often used as a measure of the strength of a linear relationship (i.e., the degree of similarity) between two sets of data in a variety of fields. However, in the field of scanning electron microscopy (SEM), it is frequently difficult to properly use the correlation coefficient because SEM images generally include severe noise, which affects the measurement of this coefficient. The current study describes a method of obtaining a correlation coefficient that is unaffected by SEM noise in principle. This correlation coefficient is obtained from a total of four SEM images, comprising two sets of two images with identical views, by calculating several covariance values. Numerical experiments confirm that the measured correlation coefficients obtained using the proposed method for noisy images are equal to those for noise-free images. Furthermore, the present method can be combined with a highly accurate and noise-robust position alignment as needed. As one application, we show that it is possible to immediately examine the degree of specimen damage due to electron beam irradiation during a certain SEM observation, which has been difficult until now.

## 1. Introduction

The general-purpose scanning electron microscope is used to observe a variety of surface structures in individual specimens under various operating conditions (i.e., operational parameters, such as the accelerating voltage, incident current, pressure, scanning mode, working distance, magnification, and detector). These operational parameters are determined as appropriately as possible to obtain a signal containing useful information. Nevertheless, the magnitude of the scanning electron microscopy (SEM) signal is often inadequate for purpose; i.e., the signal can be noisy and blurred to varying degrees. The signal-to-noise ratio (SNR) is sometimes measured in SEM focusing tasks [1] and image quality evaluation [2, 3]. Other methods of checking the signal quality include the use of a Fourier transform [4, 5].

As another serious problem in SEM observation, the images may be adversely affected by radiation damage, contamination, and charging phenomena. These effects have been suppressed to some extent by various improvements in SEM equipment. However, because the extent of these effects depends on the characteristics of particular specimens, various types of degradation of the specimen surface structure must be carefully monitored during SEM observation. This evaluation is usually done by visual inspection, and it is a difficult task even for a skilled SEM operator. It would be useful to be able to quantify the situation of change (i.e., damage) for each specimen during SEM observation in a practical way. Such quantification is the main objective of our study.

For the above purpose, we consider using Pearson's correlation coefficient. This coefficient is conveniently used as a measure of the similarity of two images in a comparison of each pixel value between the two images and quantifies the relationship over a scale of 1 to −1 (where a value of 1 indicates a perfect match and a value of zero indicates no relationship). There are several measures for the degree of correlation in data, but Pearson's correlation coefficient is the best-known and most commonly used correlation coefficient in determining the similarity of images. Hence, in this paper, we mainly discuss Pearson's correlation coefficient and simply refer to it as the correlation coefficient (CC). (Naturally, the CC is used not only in the field of image processing but also frequently in a wide range of fields.)

The CC and other metrics have long been used in the field of SEM for template matching and similar tasks [6, 7]. Even today, advanced methods based on the principles of the CC are used in a variety of studies that require the alignment of SEM images [8–10]. In addition, a modified form of the CC has been used in resolving data asynchronicity (i.e., the time difference in the data acquisition) in the field of high-speed atomic force microscopy [11, 12].

Unfortunately, the measured CC is always affected by noise. Moreover, the noise amplitude usually varies among microscopy images. Specifically, compared with the true value of the CC obtained from a noise-free image, the measurement disturbed by noise will be considerably smaller than the amplitude of the noise. This problem has to be solved because it adversely affects various studies in the field of microscopy.

A method of preventing failures in image integration resulting from severe noise when using the CC has already been discussed in the field of cryo-electron microscopy [13]. The CC is frequently used in fluorescence microscopy, and the reliability of colocalization detection has been improved by applying to the CC a factor that corrects for the adverse effect of noise [14, 15].

However, severe noise in an SEM image interferes with the measurements of the CC, and it remains difficult to numerically examine the degree of specimen damage due to electron beam irradiation during a certain SEM observation. To settle this problem, an intuitive and easily understandable method of evaluating sample degradation should be as unaffected by SEM noise as possible, even under extremely noisy conditions. In this paper, we study a useful technique that further improves the robustness of the CC against noise and fully satisfies this requirement.

## 2. Method of Obtaining CC That Is Largely Unaffected by the SEM Noise Component

The relationship between two variables can be confirmed by creating a scatter plot, i.e., a plot of the values of the two variables against each other. Scatter plots are frequently used in various fields of research. The present paper targets two images (having pixel value data) with fairly high similarity, and the images are thus linearly related to each other. This linearity can be assessed by checking that the scatter plot yields a relatively straight line. Examples of linear relationships between simulated images are shown later. CC is used to numerically and intuitively evaluate the degree of the linear relationship (similarity) in a scatter plot.

In the present study, CC is used to measure the statistical relationship between two images (*I*_1_, *I*_2_). It is based on the covariance (*Cov*(*I*_1_, *I*_2_)), which gives the similarity of the two images, as shown in equation (1). This formula for measuring CC comprises the covariance of the two images and the variances (*Var*(*I*_1_), *Var*(*I*_2_)) for each image. Specifically, the CC is a measure of the similarity of an image (*I*_2_) to a reference image (*I*_1_). The measured value of CC is expected to be close to 1 if the two images have identical views and close to zero if the two images have totally different views as mentioned above [6].

(1) $$\begin{array}{c} \text{CC}=\frac{Cov\left(I_{1},I_{2}\right)}{\sqrt{Var\left(I_{1}\right)}\;\sqrt{Var\left(I_{2}\right)}} \end{array}$$

CC is often used ignoring the effect of the noise component in the image data. However, this noise component is a potential weakness because it disturbs CC measurements more often than expected, and it prevents the use of CC in various applications [13].

**Figure 1:**
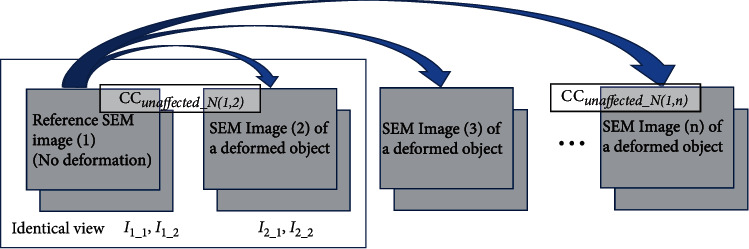
A procedure for obtaining CC_unaffected_*N*_ calculated by equation (2), which uses a total of four images shown by the large rectangular frame. CC_unaffected_*N*_ relative to the reference image is calculated as many times as required.

We propose a correlation coefficient that is unaffected by the SEM noise component (CC_unaffected_*N*_) to address the above problem in a fundamental manner. CC_unaffected_*N*_ is measured for a total of four images as shown by the large rectangular frame in Figure 1. Specifically, two reference SEM images with identical views (*I*_1_1_, *I*_1_2_) and two other SEM images with identical views (*I*_2_1_, *I*_2_2_) including the object deformed by, for example, electron beam irradiation are required, as shown in equation (2). We replace the variance values used in equation (1) with covariance values, because the SEM signal includes the desired signal and noise, and its variance values contain both influences. In other words, we can obtain the standard deviation of the desired signal only without the effect of the noise component by calculating the square root of the covariance of two images with identical views [16, 17]. The noise differs among the four images (it is necessary that noise included in the four images is independent):

(2) $$\begin{array}{c} {\text{CC}}_{\text{unaffected}\_N}=\frac{Cov\left(I_{1_{\text{ave}}.},I_{2_{\text{ave}}.}\right)}{\sqrt{Cov\left(I_{1\_1},I_{1\_2}\right)}\;\sqrt{Cov\left(I_{2\_1},I_{2\_2}\right)}} \end{array}$$

Here, *I*_1_ave_._and *I*_2_ave_._, respectively, denote the integrated image of *I*_1_1_ and *I*_1_2_ (obtained by averaging the two images with identical views) and that of *I*_2_1_ and *I*_2_2_. This averaging is necessary to suppress errors in measurements when using images with an insufficient number of pixels and a low SNR. This is essentially equivalent to the abovementioned method of applying a coefficient to the CC that compensates for the adverse effects of noise. Similarly, the concept of the correction coefficient works well for Spearman's rank correlation, which is not treated in this study [14]. However, from the viewpoints of the ease of use and ease of understanding, this study does not use a correction factor but rather newly proposes an expression in which the variance values of equation (1) are simply replaced by the covariance values (equation (2)) and used for the SEM images. CC_unaffected_*N*_ is calculated with respect to the reference image as many times as required, as shown in Figure 1.

To obtain *I*_1_1_ and  *I*_1_2_ with identical views, an odd-looking SEM image with an incorrect aspect ratio is acquired by scanning each line twice. Two images with the correct aspect ratio are then reconstructed by separating out lines properly from the SEM image. The same procedure is used to acquire *I*_2_1_ and  *I*_2_2_. Images obtained through this scanning provide nearly perfectly identical views [3, 18]. It is not technically difficult for many modern SEMs to acquire these images. If SEM images with identical views are obtained by normal scanning, it is highly recommended to acquire the images under conditions for which sample drift can be ignored, e.g., conditions of low magnification or rapid scanning. We used an appropriate rapid scanning mode of 0.5 s/image in an experiment for which results are shown later. In some cases, position alignment (which is a pattern matching technique) [10] should be conducted for the images before the calculation of CC_unaffected_*N*_.

**Figure 2:**
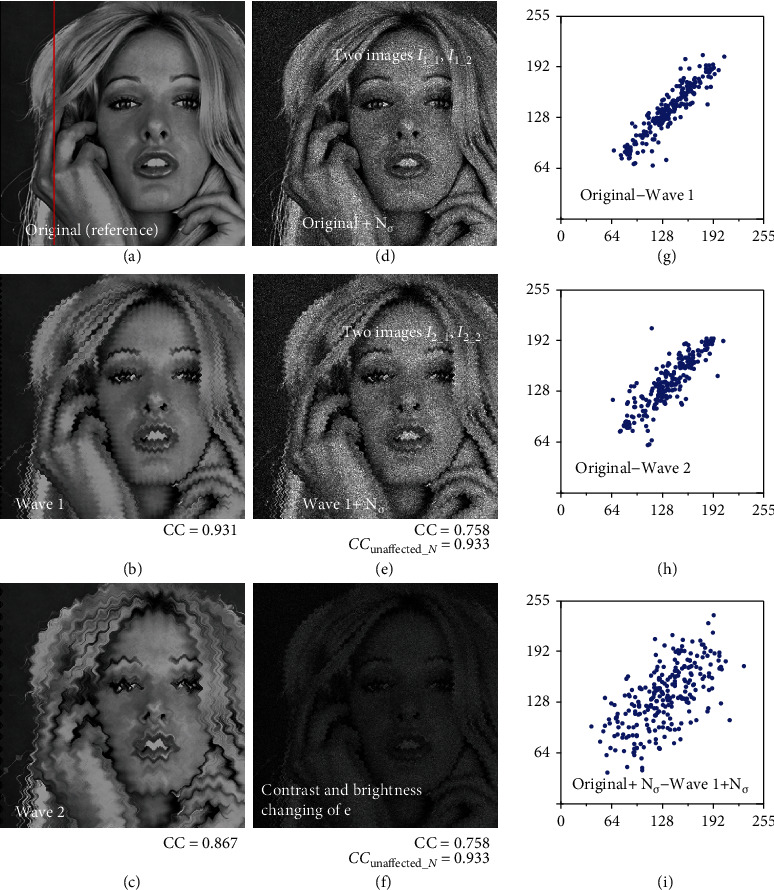
A simulation for confirming the accuracy of the CC_unaffected_*N*_ measurement: (a) original (reference) image; (b) intentional object deformation using an image-processing technique that introduces waves of strength 1; (c) intentional object deformation using an image-processing technique that introduces waves of strength 2; (d, e) results of adding Gaussian white noise to (a) and (b), respectively; (f) image (e) with reduced brightness and contrast; (g, h) scatter plots comparing the reference image (a) and each deformed image (b and c); (i) scatter plot comparing (d) and (e). See the main text for details.

A simulation is performed to confirm the accuracy of the method proposed for the measurement of CC_unaffected_*N*_.  Figure 2(a) is a single original image without deformation of the object and noise (the image is widely used in testing). The image has 256 × 256 pixels and an 8-bit quantization. Two intentionally deformed (degraded) images (Figures 2(b) and 2(c)) are created from the original image through image processing that introduces waves of strength 1 and strength 2 throughout the original image. The scatter plots for the relations between the reference image (Figure 2(a)) and each deformed image (Figures 2(b) and 2(c)), with no noise yet having been added, are shown in Figures 2(g) and 2(h). Data in the scatter plot are limited to the data on the red line drawn in Figure 2(a) so that the amount of data in the plot is visually appropriate. The variation of data points from linearity in the scatter plot (Figure 2(h)) for the image deformed by waves with strength 2 (Figure 2(c)) is larger than that in the scatter plot (Figure 2(g)) for the image deformed by waves with strength 1 (Figure 2(b)). However, the shapes of the scatter plots are similar. This indicates that the rough shape of the object remains well preserved. Corresponding to the situation of gradually increasing deformation of the object and expanding the variation of data in the scatter plot, the CC relative to the reference image (calculated for the whole image using equation (1) and shown next to each image (0.931, 0.867)) is slightly smaller than 1, as expected. In other words, when there is no noise, we can assume that CC is measured properly.

We next add Gaussian white noise with a standard deviation of 20 to the images in Figures 2(a) and 2(b). A noisy image is thus prepared for each image as shown in Figures 2(d) and 2(e). The scatter plot for the relation between the noisy reference image (*I*_1_1_ in Figure 2(d)) and noisy deformed image (*I*_2_1_ in Figure 2(e)) is shown in Figure 2(i). Compared with the scatter plot in Figure 2(g) (no noise), the variation in data from linearity in the scatter plot of Figure 2(i) is considerably larger owing to the adverse effect of the noise. Additionally, the variation from linearity in this scatter plot is larger than that in the scatter plot in Figure 2(c), where there is no noise and only stronger deformation (strength 2). It is therefore difficult to accurately discuss the degree of image deformation using a scatter plot when there is severe noise. Naturally, the value of CC (0.758) relative to the noisy reference image (Figure 2(d)) is considerably reduced, as expected, because of the addition of noise.

The situation changes completely when the newly proposed CC_unaffected_*N*_ (equation (2)) is applied to the four images in Figures 2(d) and 2(e). The values of CC_unaffected_*N*_ (0.933) nearly match CC (0.931) in the case of images without noise. It is now no longer necessary to be concerned about SEM noise when using the correlation coefficient (it will be shown later that CC_unaffected_*N*_ can also be calculated correctly for several other noise levels. Not surprisingly, changing the contrast and/or brightness of the image using image processing techniques as shown in Figure 2(f) does not change CC or CC_unaffected_*N*_ (pixel values in Figure 2(f) are exactly half those in Figure 2(e)). This is one of the original advantages of CC, which CC_unaffected_*N*_ also possesses. This advantage would be useful in SEM because the contrast and brightness of SEM images are frequently adjusted or change unstably, e.g., when the strength of the incident electron beam fluctuates. The usefulness of this type of technique under a wider range of noise conditions has been shown in a previous study using the correction factor mentioned above [14].

## 3. Measurement of the Deformation (Damage) State of a Specimen during SEM Observation Using *CC*_*unaffected*_*N*_

Specimens are damaged by electron beam irradiation during SEM observations to varying degrees. In particular, the surface structure of a sample containing moisture may be drastically deformed by moisture evaporation or heating damage, even if a low-vacuum mode is used. In this section, we show that it is possible to examine the degree of sample deformation (i.e., damage), which was difficult to measure numerically until now, during SEM observations.

Digital SEM signals output from a general-purpose scanning electron microscope (S-3400N, Hitachi High Technologies, Tokyo, Japan), which had a low-vacuum mode coupled with a cooling stage (Deben UK, Ltd.), were used in obtaining two SEM images with identical views as described above. In addition, SEM digital video signals were continuously and rapidly acquired using a personal computer controlled with LabVIEW software (National Instruments, Austin, TX, USA). To obtain better results, the personal computer was equipped with a DVI3USB 3.0 video grabber for lossless video capture from a device with a digital visual interface output port (Epiphan Systems Inc., Ottawa, Ontario, Canada).

### 3.1. Changes in CC_unaffected_*N*_ Measurements When an Electron Beam Is Irradiated for a Relatively Long Period under a Low-Vacuum Condition

In the experiment, CC_unaffected_*N*_ was measured under SEM conditions of a voltage of 15 kV, a pressure of 100 Pa, a magnification of 350x, the use of a (semiconductor-type) backscattered electron detector (BSED), a working distance of 6.6 mm, 640 × 480 pixels, an acquisition time of 10 s/image, and a system that provides cooling at −10°C. The specimen used in the experiment was a fresh leaf of Japanese shiso lettuce (*Perilla*).

**Figure 3:**
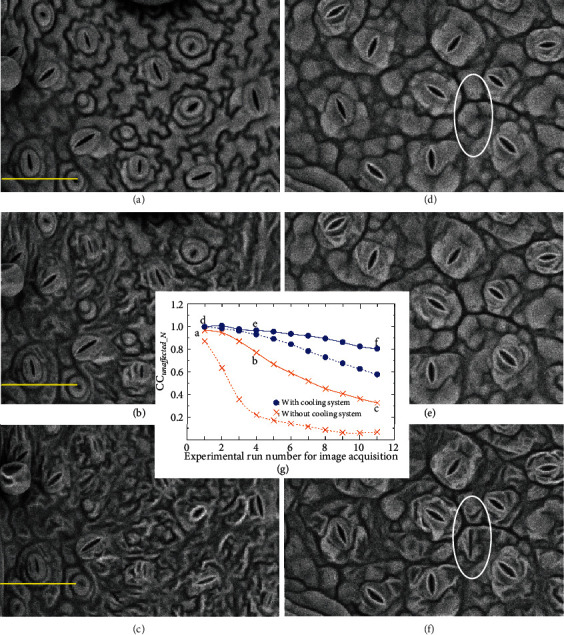
Changes in CC_unaffected_*N*_ measurements when an electron beam is irradiated for a relatively long period under a low-vacuum condition. (a–c) Without the cooling system, the acquisition of two SEM images with identical views of a fresh leaf of Japanese shiso lettuce (*Perilla*) was obtained (for a total acquisition time of 10 s + 10 s = 20 s) 12 times continuously (for a total electron beam irradiation time of 240 s). Examples of SEM images are the first (a), fifth (b), and last (c) images taken. (d–f) Images obtained with the cooling system. The image acquisition procedure for (d–f) is the same as that for (a–c). (g) Measurement results of CC_unaffected_*N*_. See the main text for details.

First, without the cooling system, two SEM images with identical views were acquired (for a total acquisition time of 10 s + 10 s = 20 s) 12 times continuously (for a total electron beam irradiation time of 240 s) with as little time as possible between each image acquisition (although two SEM images with identical views were required at each measurement point considered in this study, not surprisingly there was no wasted effort because an image with a $\sqrt{2}$ times higher SNR can be produced by averaging the two images afterwards). Figures 3(a)–3(c) show example SEM images, i.e., the first (reference image), fifth, and last images. We see that the deformation of the surface structure of the specimen by the electron beam irradiation becomes increasingly worse. Twice-expanded images are presented to show the change clearly (Figures 3(a)–3(f)). Here, the deformation includes the drift of the sample. A comparison of the positions of the three yellow bars in Figures 3(a)–3(c) shows that the serious specimen drift is mainly upward at this time.

We next reduced the sample deformation using a cooling system. The image acquisition procedure for Figures 3(d)–3(f) was the same as that for Figures 3(a)–3(c) except that the cooling system was turned on. Compared with the results in Figures 3(a)–3(c), the results in Figures 3(d)–3(f) show a considerable improvement in specimen deformation, although the final image (Figure 3(f)) showed damage to a certain degree (there was also only a slight specimen drift, which was not so visible). In particular, there is little visual difference between Figure 3(d) (i.e., the reference image) and Figure 3(e).

Now that the visual evaluation of the difference in sample damage with and without a cooling system is complete, we calculate CC_unaffected_*N*_ for the reference image and each image of the deformed specimen and plot the results as the solid lines in Figure 3(g) (where a–f indicate the measurements obtained from Figures 3(a)–3(f), respectively).

It is noted that the calculated value is not the simple CC_unaffected_*N*_ but the value of CC_unaffected_*N*_ obtained after performing a position alignment based on the zero-mean normalized cross-correlation [10], which is frequently used in pattern matching techniques and generally yields stable results. Here, because the SEM images in Figure 3 are twice-expanded images, the area used for alignment and CC_unaffected_*N*_ measurements was set at 320 × 240 pixels. Without the position alignment, the values of CC_unaffected_*N*_ would be meaningless in this experiment and far from the true values, which are indicated by the dotted lines of the same color, owing to the specimen drift mentioned earlier. This is true even for slight specimen drift, which was not so visible, as shown in Figure 3(f) (this additional process is not necessary if the effect of specimen drift is important in the experiment). Note that there was no specimen drift in the simulation of Figure 2, and its effect on CC_unaffected_*N*_ is discussed here for the first time. The required accuracy of alignment depends on what purpose CC_unaffected_*N*_ is used for. In this study, we performed alignment with an accuracy of 1 pixel. In some cases, a more accurate method of alignment may be needed, but we have obtained enough useful information from the experimental data in the present study using the alignment method described above. To handle more severely deformed and different types of images, image registration—the process of estimating an optimal transformation between images (including techniques for detecting feature points and finding corresponding pairs)—has been adopted in many studies in other fields [19–22]. Most recent research on image registration has focused on the use of deep learning for feature extraction [23, 24]. However, it is not necessary to introduce this work here, because the image data considered in this study were obtained from the same detector (i.e., the BSED).

The values of CC_unaffected_*N*_ for measurement points a (without cooling) and e (with cooling) in Figure 3(g) are the same (0.966), and it is thus assumed that the degradation states are similar to each other at these points. Even though measurement point a is the first measurement, its value of CC_unaffected_*N*_ is a little lower than 1. The reason may be that four SEM images including the reference image (two images with identical views) are used in measuring CC_unaffected_*N*_ at measurement point a, without a cooling system. The value suggests that subtle specimen deterioration, which does not affect the specimen observation, has already occurred. In comparison, the CC_unaffected_*N*_ measurements show that a cooling system would allow the acquisition of two SEM images with identical views at least several times without serious specimen degradation (the CC_unaffected_*N*_ values remain close to 1 for a certain period of time). However, it is noted that even though a cooling system is used, a false surface structure (which might be mistaken for stomata) can sometimes form after electron beam irradiation for a long period, as shown in the white ellipse in Figure 3(f) (for comparison, the white ellipse is also drawn in Figure 3(d)). The CC_unaffected_*N*_ value in Figure 3(f) is considerably reduced to 0.803.

From the discussion so far, we can numerically determine the period of time (CC_unaffected_*N*_ ≈ 1) when little or no sample degradation has occurred in SEM observations under various circumstances. However, the degree of change in the measured correlation coefficient will change depending on the properties of the sample (e.g., the robustness of the surface structure to electron beam irradiation) and the SEM conditions (e.g., the instrumental magnification and vacuum pressure). Hence, at present, we do not know to what extent CC_unaffected_*N*_ can reduce to prevent valid observations, because it depends on the content of each SEM observation. It would be difficult to uniformly determine a single lower limit for CC_unaffected_*N*_.

A graph of CC is not shown here. The SEM images in Figure 3 do not contain much noise, and the adverse effect of noise is thus not severe. The serious effect of noise is examined in the next section.

### 3.2. Obvious Differences between CC_unaffected_*N*_ and CC Measurements for SEM Images Acquired by Rapid Scanning

**Figure 4:**
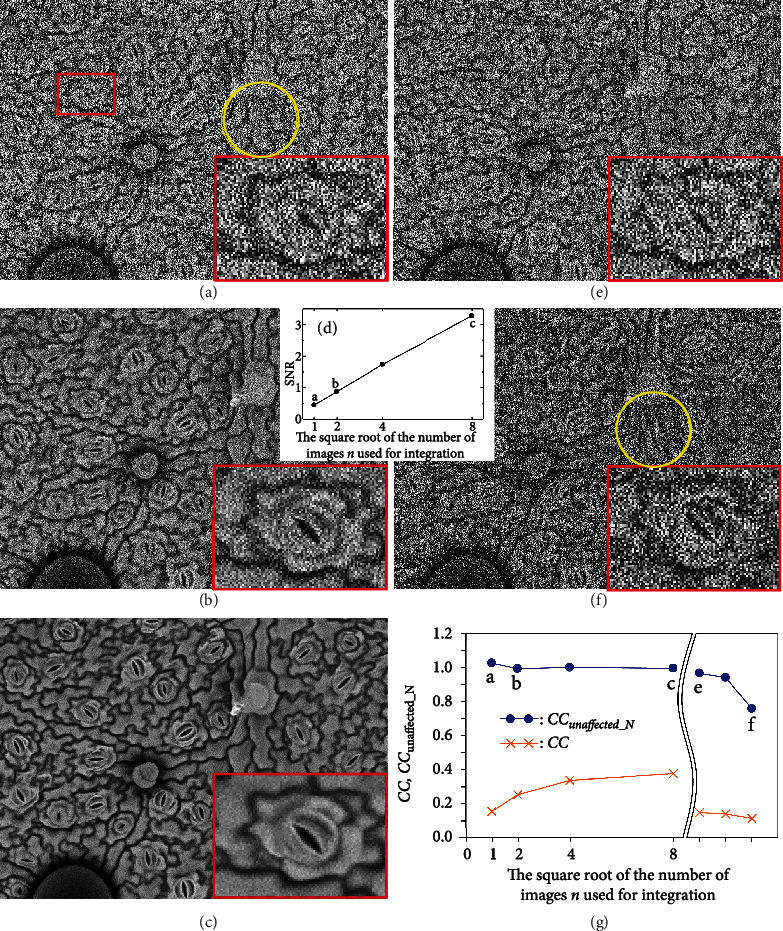
Obvious differences between CC_unaffected_*N*_ and CC measurements for SEM images acquired by rapid scanning (at an acquisition time per image of 0.5 s). (a, e) First image (reference image) and last image in a series of noisy SEM images obtained by continuous acquisition. (b) Integrated image of four SEM images. (c) Integrated image of 64 SEM images. (d) Measured SNR improvement due to image integration. (f) Additional (deformed) SEM image acquired after a long period of electron beam irradiation. (g) Comparison between measurements of CC_unaffected_*N*_ and CC. See the main text for details.

The discussion in the previous section revealed that when a cooling system was used, both deformation and drift in the specimen were negligible for a certain period of time in the observations made under the above-mentioned conditions. Within that period of time, a series of SEM images (130 images, 65 pairs) were continuously and rapidly acquired under the same conditions of Figures 3(d)–3(f) except for the adoption of a scanning time of 0.5 s per image. These images were considered to have identical fields of view (in fact, 512 SEM images (i.e., 256 pairs) were acquired and the first 130 images were mainly used in the experiments). The scanning speed was 20 times that for Figure 3 and all images are thus seriously noisy (see Figure 4(a) and the enlargement of the area in the red rectangle). However, unlike the images in Figure 3, the images in Figure 4 are not only noisy but also basically unexpanded, and the SEM images may thus look fairly different (all images in Figure 3 were twice expanded to clearly show the difference in specimen deformation). Incidentally, a scan speed higher than this (e.g., the speed of a TV scan) was not adopted in this study owing to the poor frequency characteristics of the detector (i.e., the BSED) [25].

The purpose of this experiment is not only to compare CC_unaffected_*N*_ and CC but also to numerically display at short time intervals (e.g., 1 s) under noisy conditions the changes in the situation of specimen degradation, which are difficult to confirm. From the discussion so far, CC_unaffected_*N*_ is expected to be close to 1 (which is evidence of no sample deformation) and measured CC is expected to be considerably small owing to noise. To experimentally demonstrate the robustness of CC_unaffected_*N*_ against noise, 1 (i.e., the reference image, Figure 4(a)), 4 (Figure 4(b)), 16, and 64 (Figure 4(c)) integrated images are created from the 130 SEM images (65 pairs) such that we have several SEM images with different levels of noise.

The main image and expanded image in Figure 4(a) (without integration) are visually noisy. The SNR is calculated as 0.442 using the following method. We need two images with identical views (*I*_1_1_, *I*_1_2_) for each integrated image to measure the SNR, which is inversely proportional to the noise amplitude, and the process thus uses up to 130 images. The SNRs of the integrated images are calculated using equation (3) [2, 3]. It is noted that this equation looks similar to equations (1) and (2) in form but has different content:

(3) $$\begin{array}{c} \text{SNR}=\sqrt{\frac{Cov\left(I_{1\_1},I_{1\_2}\right)}{\sqrt{Var\left(I_{1\_1}\right)\cdot Var\left(\;I_{1\_2}\right)}-Cov\left(I_{1\_1},I_{1\_2}\right)}} \end{array}$$

Not surprisingly, in proportion to the square root of the number of integrated images *n*, the SNR theoretically improves (i.e., there is a noise reduction), in agreement with experiment as shown in Figure 4(d).

Now that several integrated SEM images with different SNRs have been obtained, the measured values of CC_unaffected_*N*_ and CC relative to the reference image are obtained and plotted as shown in Figure 4(g) (where a–c indicate the measured values obtained from Figures 4(a)–4(c), respectively). The horizontal axis gives the square root of the number of images used for integration. The first measurement of CC_unaffected_*N*_, at measurement point a, is obtained, using equation (2), from the reference images (Figure 4(a) (*I*_1_1_, *I*_1_2_) and the next images (*I*_2_1_, *I*_2_2_), which are also not integrated (these images are not shown in Figure 4). Despite the various noise conditions, all CC_unaffected_*N*_ values are close to 1, as theoretically expected, because, as we know from the previous discussion, there is no sample deformation during this period. It is noted that even though the SNR of the reference image and the degraded image are different, there is no problem in calculating CC_unaffected_*N*_ using equation (2), owing to the properties of covariance. Incidentally, the value of CC_unaffected_*N*_ never exceeds 1 theoretically but sometimes does slightly in practice owing to measurement errors in the covariance. The value of CC_unaffected_*N*_ for measurement point a in Figure 4(g) (i.e., a very noisy image) is 1.025, which is the largest value we have observed so far. The error may be able to be suppressed by increasing the number of pixels used in the calculation; however, we believe that it is not necessary to discuss this small error further in this section.

In contrast with the excellent results of the CC_unaffected_*N*_ measurements, it is confirmed that CC is not useful owing to the effect of severe noise, as depicted in Figure 4(g). Specifically, the CC results give false information that the sample damage is severe, and moreover, the degree of the false information depends on the level of noise. Even if we could use an integrated image with no noise at all, the effect of the noise in the reference image is severe, and we find that CC cannot exceed 0.4 in this experiment when we apply this condition to equation (1).

As an additional experiment, CC_unaffected_*N*_ and CC were measured for SEM images of the 65th (Figure 4(e)), 120th, and 240th pairs (Figure 4(f)) without integration and added to the graph (Figure 4(g)). To the right of the double wavy lines, the horizontal axis no longer gives the square root of the number of images used for integration; i.e., the number of images used for integration is always 1 (without integration). Measurement points e and f correspond to Figures 4(e) and 4(f), respectively. Figure 4(e) represents the last pair of the 130 images (65 pairs) mentioned above. CC_unaffected_*N*_ remains close to 1 because there was almost no specimen deformation. Meanwhile, CC_unaffected_*N*_ values for the 120th and 240th pairs of images are affected by the electron beam irradiation for considerably longer time than the value for the 65th pair used in this experiment up to now. Here, because these two values of CC_unaffected_*N*_ were obtained after performing a position alignment, similar to the procedure in the case of Figure 3, they are reliable values indicating only the effect of specimen deformation (to match the measurement conditions in Figure 3, an area comprising 320 × 240 pixels near the center of Figure 4 was used for all measurements). In Figure 4(f) (240th pair, where the 120th pair is not shown in Figure 4), the surface structure in the yellow circular frame is greatly degraded (where the yellow circular frame is also drawn in Figure 4(a) for comparison), and the CC_unaffected_*N*_ value relative to the reference image (as measured for the whole image) is considerably reduced as expected, though the situation of specimen deformation seems to be locally different (if we had measured CC_unaffected_*N*_ at each location in the partial images, we may have obtained different values). Incidentally, a comparison of images shows a slight decrease in brightness for Figure 4(g), but this does not have any effect, as shown in Figure 2.

Electron beam irradiation was performed for a total of 240 s before the image in Figure 4(f) was acquired. This irradiation time is equivalent to that for the image in Figure 3(f) in the previous section. We can therefore compare the two images. However, after the experiment of Figure 3, because that of Figure 4 was performed without replacing the specimen, the specimen condition (i.e., the period that the sample was kept under the vacuum condition) was different between the experiment of Figure 3 and that of Figure 4. Under such circumstances, CC_unaffected_*N*_ values for Figure 3(f) (0.803) and Figure 4(f) (0.757) were similar to each other, although the latter was slightly inferior. This may be a reasonable result (without electron beam irradiation, the degradation of SEM specimens simply placed under low-vacuum conditions with the cooling system would not be as severe as expected). Unfortunately, it is impossible to suitably compare the visual differences in specimen degradation because the level of noise differs too much between the two images.

The proposed method worked well even under the noisy conditions shown in Figure 4 (SNR of 0.442, measurement area of 320 × 240 pixels). This result may be sufficient for the general use of SEM. However, because our method is expected to be used in SEM observations where low-dose and/or very weak SEM signal conditions, like those of a low vacuum, are applied to suppress sample degradation, we would like to obtain continuous and stable measurement results even under poorer conditions. Unfortunately, the previous study using the correction factor [14] simulated that the variability of measurements becomes unacceptable when the noise increases beyond a certain level. An effective solution to this problem is needed.

We usually need huge data to obtain an accurate measurement of CC_unaffected_*N*_ at a much lower SNR because the measurement of covariance used in the calculation is sometimes unstable for certain images having an insufficient number of pixels. If huge data cannot be employed for the CC_unaffected_*N*_ calculation for various reasons (e.g., the deformation of the monitoring specimen in the above-mentioned adverse environment), we can systematically apply a simple technique to improve the image quality along the lines of the following equations [3, 26].

(4) $$\begin{array}{c} \frac{E\left\{Cov_{s}\;\left(I_{1},I_{2}\right)\right\}}{\sqrt{Var\left(Cov_{s}\;\left(I_{1},I_{2}\right)\right)}}=\sqrt{number\;\text{of }pixels}\frac{SNR^{2}}{\sqrt{2SNR^{2}+1}} \end{array}$$

Here, E{} is the statistical expectation value. Equation (4) includes the lesser-known potential usefulness of the noise immunity for SEM. The numerator and denominator on the left-hand side, respectively, show the desired signal and the standard deviation of the aggregation of many sample covariance (Cov_s_) values. In other words, the left-hand side is an index of the theoretical scattering of measurements of covariance in the case of a sample of a certain size. A larger value of this index corresponds to a better result for the covariance measurement. To improve the numerical value of the index, we can adjust the SNR and the number of pixels of the sample (SEM image) on the right-hand side of equation (4). When the acquisition time of an SEM image is constant, the SNR is strongly related to the number of pixels. Concretely, if the number of pixels decreases by a factor of 4, the SNR increases by a factor of 2 regardless of the noise distribution. After image acquisition, the reduced image can be attained by averaging over an area of 2 × 2 pixels (if the desired signal does not satisfy sufficiently the sampling theorem, the degree of improvement in the SNR may be reduced to some extent). According to equation (4), the measured value of a stable covariance is obtained by improving the SNR rather than by increasing the number of recording pixels. However, it is noted that this contrivance is effective only for data acquired under low-SNR conditions.

**Figure 5:**
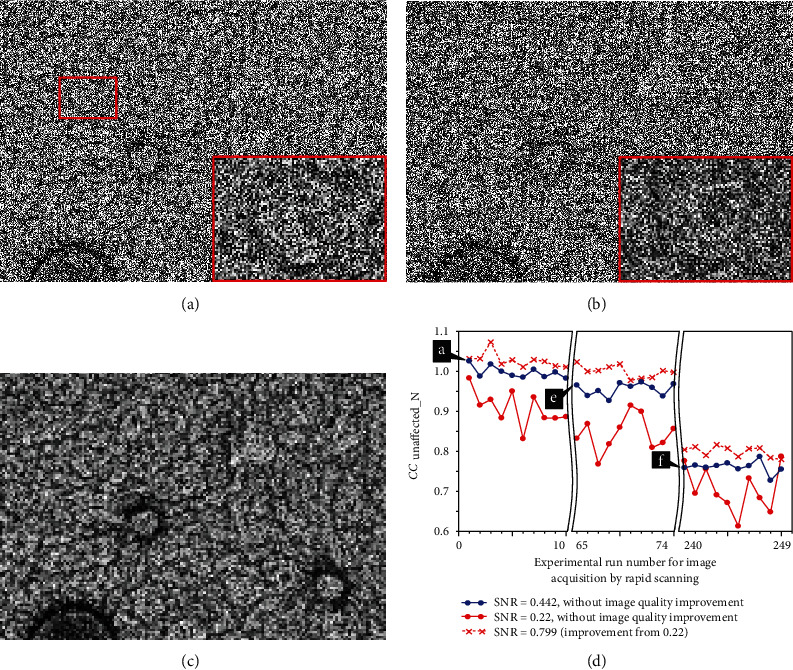
Simulation confirming the effectiveness of the CC_unaffected_*N*_  measurement under extremely noisy conditions. A series of simulated images were generated by superimposing Gaussian white noise on the 512 SEM images (256 pairs) acquired for Figure 3. (a, b) Very noisy simulated images corresponding to Figures 3(a) and 3(f), respectively. (c) Processed image for Figure 5(a) with an improved SNR. (d) Graph showing that the proposed image processing technique sufficiently suppresses CC_unaffected_*N*_fluctuations. See the main text for details.

The experiment for which results are shown in Figure 5 was performed to demonstrate the effectiveness of the proposed technique. As experimental data, a series of simulated images with an SNR of 0.22 (almost half that in Figure 4(a) (rapid scanning)) were produced by superimposing Gaussian white noise on 512 noisy SEM images (256 pairs) acquired for Figure 4 (in Figure 5, 30 of the 256 pairs are used to create a graph). The simulated images corresponding to the above-described Figures 4(a) and 4(f) are, respectively, shown in Figures 5(a) and 5(b). Unsurprisingly, the surface structures in the two images are so heavily disturbed by noise that it is almost impossible to visually distinguish the difference between them.

In discussing CC_unaffected_*N*_ calculated from the simulated images and the improvement in the scattering of the measurements, the measurement points a, e, and f in Figure 4(g), which are CC_unaffected_*N*_ values measured for SEM images of the first pair (Figure 4(a), SNR of 0.442), 65th pair (Figure 4(e)), and 240th (Figure 4(f)), are again shown in Figure 5(d). The series of measurement points following each point (30 points in total) is shown in Figure 5(d) (blue solid line). It is noted that the data in the graph are discontinuous at the two double wavy lines. As expected, the variation at these measurement points is sufficiently small. We thus confirm in more detail that the system is capable of detecting the deformation of the sample properly under the conditions of Figure 4 (from measurement point f, CC_unaffected_*N*_ measurements show severe sample degradation). Note that the standard deviation from the first point (first pair) to the 20th point (74th pair), where the sample has not yet deformed, is 0.0263.

For the series of simulated images with an SNR of 0.22 (with noise added), the variation in the CC_unaffected_*N*_ measurement points is large (having a standard deviation of 0.0534), as expected. It is thus difficult to find a feature in the CC_unaffected_*N*_ graph (red solid line) that should be small after the 21st point (240th pair) owing to sample deformation. When applying a 4 × 4 pixel average according to equation (4) (for improvement of the image quality), a series of smaller images comprising 75 × 55 pixels with a higher SNR can be used to compute CC_unaffected_*N*_. The processed image for Figure 5(a) with an improved SNR of 0.799 is shown in Figure 5(c). The graph of CC_unaffected_*N*_ (red dotted line) obtained from those images (including Figure 5(c)) shows sufficiently suppressed fluctuations (with a standard deviation of 0.0215), and we can therefore detect the sample degradation reliably at a glance under the high-noise noise condition.

There are few options for deciding the degree of pixel averaging. For now, the degree of pixel averaging is decided by trial and error according to the improvement in the CC_unaffected_*N*_ variation and the SNR within the range where the desired signal is not attenuated much by averaging. In some cases, this process of improving the image quality may result in CC_unaffected_*N*_ being slightly larger than the true values. However, this does not affect the usefulness demonstrated in Figure 5. In summary, the discussion presented in this section suggests that CC_unaffected_*N*_ can be used to measure the degradation state of SEM specimens numerically and successively under severely noisy operating conditions.

## 4. Conclusions

A method of calculating a correlation coefficient that is unaffected by SEM noise in principle, which is easily understandable and easy to use, was proposed. This correlation coefficient indicates the accuracy of measurements under several noisy conditions of SEM. The correlation coefficient, CC_unaffected_*N*_, is obtained from four SEM images, comprising two sets of two images with identical views, by calculating several covariance values. In addition, a reliable image processing technique, which is even applicable to poorer SEM conditions, is used in combination with CC_unaffected_*N*_ to obtain sufficiently stable measurement results. As a particularly useful application, CC_unaffected_*N*_ can be combined with rapid scanning to examine the degree of specimen damage numerically and successively under a low-vacuum condition (i.e., a severely noisy operating condition), which has been difficult until now. In the near future, CC_unaffected_*N*_ is expected to have a wide range of applications, including its use as an important indicator of the degradation state of specimens in SEM systems.

## Data Availability

The data used to support the findings of this study are available from the corresponding author upon reasonable request.
